# Catalysing global surgery: a meta-research study on factors affecting surgical research collaborations with Africa

**DOI:** 10.1186/s13643-024-02474-8

**Published:** 2024-03-18

**Authors:** Thomas O. Kirengo, Hussein Dossajee, Evans M. Onyango, Reema H. Rachakonda, Bailey Schneider, Declan P. Sela, Zahra Hosseinzadeh, Zohaib Nadeem, Nchafatso G. Obonyo

**Affiliations:** 1 Imara Hospital, Embu, Kenya; 2 MP Shah Hospital, Nairobi, Kenya; 3 Ministry of Health, Kajiado County, Kenya; 4 Faculty of Medicine, University of Queensland, Brisbane, Australia; 5 Critical Care Research Group, Brisbane, Australia; 6 KEMRI-Wellcome Trust Research Programme, Kilifi, Kenya; 7 Kenya Medical Association, Nairobi, Kenya

**Keywords:** COVID-19, Surgery, Collaborations, Research, Africa

## Abstract

**Introduction:**

In December 2019, the COVID-19 pandemic highlighted the urgent need for rapid collaboration, research, and interventions. International research collaborations foster more significant responses to rapid global changes by enabling international, multicentre research, decreasing biases, and increasing study validity while reducing overall research time and costs. However, there has been low uptake of collaborative research by African institutions and individuals.

**Aim:**

To systematically review facilitating factors and challenges to collaborative surgical research studies conducted in Africa.

**Methodology:**

A meta-research review using PubMed®/MEDLINE and Embase on surgical collaboration in Africa from 1st of January 2011 to 31st of September 2021 in accordance to PRISMA guidelines. Surgical studies by collaborative groups involving African authors and sites were included (55 papers). Data on the study period, geographical regions, and research scope, facilitating factors, and challenges were extracted from the studies retrieved from the search.

**Results:**

Most of the collaborations in Africa occurred with European institutions (76%). Of the 54 African countries, 63% (34/54) participated in surgical collaborations. The highest collaboration frequency occurred in South Africa (11%) and Nigeria (8%). However, most publications originated from Eastern Africa (43%). Leveraging synergies between high- and low- to middle-income countries (LMICs), well-defined structures, and secure data platforms facilitated collaboration. However, the underrepresentation of collaborators from LMICs was a significant challenge.

**Conclusion:**

Available literature provides critical insights into the facilitating factors and challenges of research collaboration with Africa. However, there is a need for a detailed prospective study to explore the themes highlighted further.

**Systematic review registration:**

PROSPERO 2022 CRD42022352115.

**Supplementary Information:**

The online version contains supplementary material available at 10.1186/s13643-024-02474-8.

## Background

The coronavirus disease 2019 (COVID-19) pandemic introduced a unique set of challenges in healthcare and highlighted the need for rapid collaboration, research, and interventions [1]. International research collaboration provides the capacity to respond to a multitude of rapid changes on a global scale [2]. Collaborations enable international, multicentre research, which lowers bias and increases the study power and validity while reducing overall research time and costs [3, 4]. It can influence public health by informing policy changes, promoting further research, and improving both clinical practice and patient outcomes [5, 6]. Studies conducted by large collaborative research groups tend to have higher numbers of participants and are thus more robust in their conclusions than those done by smaller groups or individuals. Additionally, international collaborative academic research is cited up to twice as much as locally authored studies [7].

Historically, there has been a long-standing low uptake of collaborative research by institutions and individuals in low- and middle-income countries (LMICs), especially in Africa, leading to siloed single-centre research [6, 8]. Additionally, LMICs have diminished research investment and capacity leading to lower volumes of first or second authorship appearances in global surgery publications compared to high-income countries (HICs) [9]. Over 80% of the world’s population resides in LMICs, and these countries bear most of the global disease burden [10]. African countries carry some of the most considerable disease burdens but produce the lowest volume of research [11, 12].

Huamaní et al. (2013) reported that most research and surgical collaborations occur between HICs, and that surgical collaborations with Africa were surprisingly low [13]. However, this can be reversed by leveraging the overall development in electronic information systems and technology to establish successful collaborations [6, 14, 15]. Researchers from LMICs often experience challenges attending international conferences, therefore missing out on opportunities to meet and collaborate with colleagues from different countries. Such challenges have been overcome by the increasing trend of virtual and hybrid conferences during the COVID-19 pandemic [10]. It is not limited to traditional methods of communication since social media platforms such as Twitter/X, Instagram, WhatsApp, and Facebook are now being used as tools for collaboration and sharing information amongst researchers [16, 17]. Social media provides a scalable unit for amplifying and advancing global collaboration and partnership in surgical research. However, Navarro et al. (2020) found that despite social media being globally accessible, the majority of the global surgery content on social media arose from the northern hemisphere. An estimated 70% arose from the United States of America (USA) and the United Kingdom (UK) [9].

Over the past few decades, there has been a paradigm shift from HICs implementing programmes in LMICs to capacity building in these regions. However, comparatively, lower importance had been placed on building such a collaborative partnership geared towards enhancing research. A shift in this thinking has been observed over the past few years [18]. The power of decision-making in global health still disproportionately lies with HICs [10]. However, the partnership between LMICs and HICs can be designed to utilize synergies and mutually benefit both parties. These relationships, especially if long-term, can be helpful in knowledge and skills transfer, training, improving research capacity in LMICs, and sharing resources. Successful collaborations require strong leadership, co-design and participation by all involved parties, adequate representation, trust, openness, and mutual respect [19, 20]. Historically, collaborations have been higher between developed HICs (global-north) and LMICs (global-south), i.e. North–South collaborations, compared to South-South collaborations. Therefore, despite the power and resource dynamics, it is essential to ensure that these collaborations maintain tangible benefits for all the parties involved. Additionally, there is room for more Southern Hemisphere collaboration, particularly within LMIC settings of Africa [20].

Despite the known benefits of collaborations, co-produced research remains limited in LMICs [21]. The rate of growth of these collaborations in Africa is unknown and undescribed in current literature. This compared to high-income countries, such as the UK, where a 2017 study by the National Surgical Research Collaborative found that 99% (238/241) of hospitals providing general surgery services had participated in at least one collaborative study. The success of collaborative general surgery studies in the UK is being replicated across multiple other specialties and countries [22]. The study aims to systematically explore the facilitating factors and challenges to collaboration in surgical research with African countries.

## Methods

We conducted a meta-research, a study on how research is carried out, as the study aimed to explore factors influencing the application of collaborative surgical research [23]. An a priori protocol was registered with the National Institute for Health Research (NIHR) International Prospective Register of Systematic Reviews (PROSPERO registration number CRD42022352115). The reporting of this review was guided by the standards of the Preferred Reporting Items for Systematic Reviews and Meta-Analyses (PRISMA) statement [24].

### Search

A comprehensive search of published studies in two indexed online databases, PubMed®/MEDLINE and Excerpta Medica Database (Embase), focusing on surgical collaboration in Africa from 1st of January 2011 to 31st of September 2021 was conducted. The search findings were supplemented by additional hand-searching, screening, and retrieval of relevant articles from the reference list of the publications that met the inclusion criteria. The search terms used in [All Fields], or [Keywords], automatically mapped to relevant [MeSH Terms], were in the following categories (“surgery”, “collaborative”, “Africa”). Search strings were developed using “AND” and “OR” Boolean operators. The full search strategy (available here: searchRxiv. CABI International. Repository, 10.1079/searchRxiv.2023.00235, 10.1079/searchRxiv.2023.00236) is detailed in Supplementary Table 1  [25, 26].

All literature identified through the initial search was first populated on Excel® (Microsoft) and screened for duplicates, which were removed. Full-text publications of the studies identified as relevant on screening were retrieved and reviewed. In addition, relevant cited articles within the retrieved manuscripts were also screened for inclusion. Next, the abstracts were compiled in EndNote® (Thomson Reuters) and screened for relevance against the inclusion criteria.

### Study inclusion and exclusion criteria

Studies were eligible for inclusion if they were collaborative research projects in the field of surgery conducted at African sites or if they included African authors. Collaborative research was defined as studies involving collaborative groups, and these were identified through authorship. The authors deemed dental surgery, obstetrics, and gynaecology to have distinct characteristics and operations that distinguished them from generalizability across the other surgical disciplines. Eligibility criteria are as follows:

(i) The methodology included a collaborative research group as part of its study design.
(ii) The objectives addressed surgical care (including surgical subspecialties, anaesthesia, and specialties without the term “surgery”, e.g. burns).
(iii) At least one African study site(s) or collaborator(s) were African.
(iv) The study stated factors affecting the collaboration.

Studies were excluded if as follows: (i) non-collaborative studies, (ii) animal studies, (iii) papers where full articles were not available in English, (iv) non-surgical care-related subject matter, (v) obstetrics and gynaecological surgery work, (vi) dental surgery related work, and (vii) studies where the full-text publications were not retrievable.

### Study selection

The initial selection process was undertaken by the primary author (T. O. K.) based on the study titles and abstracts; the eligible studies were compiled in a spreadsheet (Microsoft Excel). Further, rescreening was conducted by two independent reviewers, and in cases where there was discrepancy, a third/ senior reviewer was consulted for final adjudication. All included studies received an approval decision from at least two independent reviewers. The reasons for exclusion were noted for each of the studies not included. The selection process is presented in a PRISMA flow diagram [24].

### Data extraction

A standardized data extraction tool was developed using Microsoft Excel. It was piloted and tested to improve clarity, relevance, and consistency when used by different reviewers. Using this tool, a minimum of two independent reviewers retrieved data from selected articles. Data collection variables extracted for each retrieved publication included the author(s); date of publication; study design; year research was conducted; the name and acronym of the collaborative; list of the continent(s); African country(s) involved; size and number of hospitals, centres, and registries; number of collaborators; surgical specialty; research scope and focus; and data on the facilitating factors (and challenges) for each collaboration (Supplement Table 2).

### Data analysis

Due to the heterogeneity of the data, the collected information was further systematically examined and qualitatively analysed for the results and discussion. The absence of a pre-existing analytical framework applicable to our study necessitated the design of bespoke analytical tools, informed by the literature surveyed in the preliminary stages. Qualitative analysis involved content analysis to identify facilitating factors, challenges, limitations, and recommendations by different authors. A critical appraisal tool was utilized by a minimum of two reviewers to enable a structured analysis of each study. The data on influencing factors was thematically analysed and categorized into the following: (i) information and communication, (ii) organizational structure and design, (iii) resources, (iv) ethics, (v) networks, and (vi) other (for any additional factors that could not be grouped in the larger categories) [4]. The results from each study analysed were documented separately and presented in a descriptive table (Supplement Table 2). The reviewers took into account the generalizability of the research data, limitations of the studies, and recommendations by the authors. However, we did not assess any author bias and the quality or certainty of the information from the studies as this was beyond the scope of the review.

## Results

**Fig. 1:**
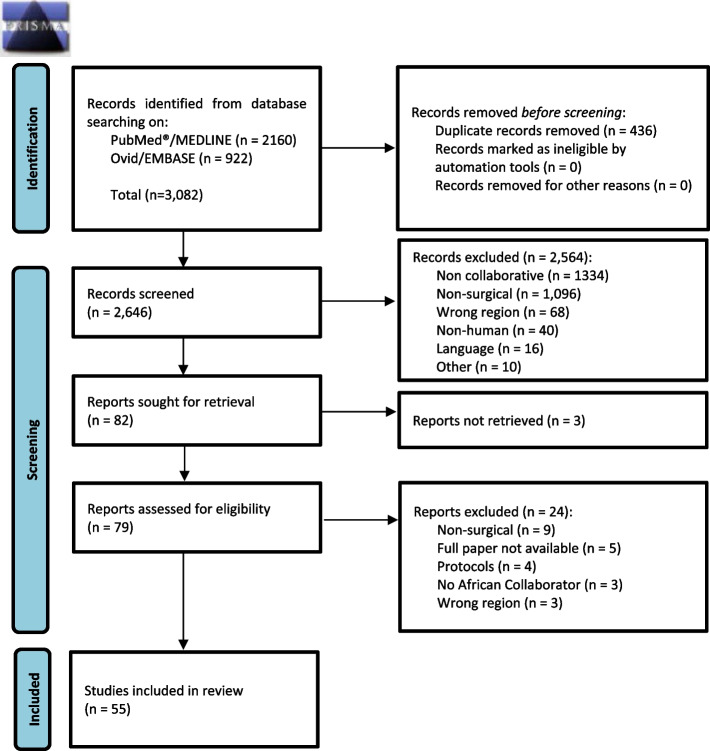
PRISMA flow diagram of collaborative surgical research studies in Africa

A total of 3082 studies were identified from a search of published literature (Fig. 1). After the initial screening, 436 (14%) duplicate studies were excluded. The abstracts of the remaining 2646 studies were compiled in EndNote and screened for relevance. A total of 2564 (83%) papers were ultimately excluded. A detailed review of the full manuscripts of the remaining 82 papers was done. Of these, 79 (3%) full manuscripts were retrievable, and three were unretrievable. Finally, 55 (2%) papers met the inclusion criteria for the meta-research review. The flow of studies retrieved from the search, excluded, and included are in the PRISMA diagram (Fig. 1).

The full results from the meta-research review are summarized in Table 1 and detailed in Supplementary Table 3 below.

**Table 1:** Studies included in systematic review of surgical collaboratives

Author & publication year	Acronym & name of collaborative^a^	African country(s) involved	Research focus & surgical specialty
Abbas et al. (2021) [27]	GICS, Global Initiative for Children’s Surgery	Nigeria, Kenya, Egypt	Impact of COVID-19 on global paediatric surgery
Alkhaffaf et al. (2021) [28]	GASTROS International Working Group, Gastric Cancer Surgery Trials Reported Outcome Standardization	Nigeria	Gastric cancer surgery General surgery
Alphonsus et al. (2021) [29]	Collaborators for EPIC2: BNP study	South Africa	Cardiovascular risk stratification in surgery Perioperative care
Beattie et al. (2021) [30]	StEP COMPAC Group, the Standardized Endpoints in Perioperative Medicine Core Outcome Measures in Perioperative and Anesthetic Care	South Africa	Perioperative cardiovascular adverse events Perioperative care
Breedt et al. (2021) [31]	AfroSurg Collaborative	South Africa, Botswana, Namibia	Surgical safety Health system management
Firth et al. (2021) [32]	Mbarara SQUAD Consortium, Surgical Quality Assurance Database	Uganda	Surgical safety General surgery, trauma, perioperative, anaesthesia
Held et al. (2021) [33]	Authorship group “Knee surgery in LRS”	South Africa	Knee surgery Orthopaedics
Kachapila et al. (2021) [34]	NIHR Global Health Research Unit on Global Surgery, ASOS Investigators, STAR Surg Collaborative, National Institute for Health Research	Algeria, Benin, Burundi, Cameroon, Congo, DRC, Egypt, Ethiopia, Gambia, Ghana, Kenya, Libya, Madagascar, Mali, Mauritius, Namibia, Niger, Nigeria, Senegal, South Africa, Tanzania, Togo, Uganda, Zambia, Zimbabwe	Lowering surgery hospital costs Health system management
Kanmounye et al. (2021) [35]	CAANS Young Neurosurgeons Committee and WFNS Young Neurosurgeons Committee Continental Association of African Neurosurgical Societies & The World Federation of Neurosurgical Societies	Cameroon, South Africa, Liberia, Côte d'Ivoire, Nigeria, Zimbabwe, Senegal, Egypt, Kenya, Rwanda, Morocco, Algeria, Ethiopia, Niger, Congo, Uganda	Neurosurgical training in Africa
Masters et al. (2021) [36]	ORCA, Orthopaedic Research Collaboration for Africa Orca Investigators	South Africa	Gunshot-related orthopaedic trauma
Mohan et al. (2021) [37]	NIHR Global Health Research Group on Neurotrauma, National Institute for Health Research	Nigeria, Ethiopia, Rwanda	Traumatic brain injury Neurosurgery, trauma surgery
Moysidis et al. (2021) [38]	The Global Consortium	South Africa, Egypt	Autologous rental transplantation Ophthalmology
Network for Peri-operative Critical Care (2021) [39]	N4PCc, Network for Peri-operative Critical care	Ethiopia	Implementation of a perioperative registry Perioperative care
Oesophago-Gastric Anastomotic Audit Collaborative (2021) [40]	OGAA, Oesophago-Gastric Anastomotic Audit Collaborative	Rwanda, Kenya, Nigeria	Mortality from esophagectomy in oesophageal ca General surgery, upper GI surgery
PaedSurg Africa Research, 2021 [41]	PaedSurg Africa Research Collaboration	Kenya, Nigeria, Ghana, Mauritania, DRC, South Africa, Burkina Faso, Côte d'Ivoire, Congo, Ethiopia, Malawi, Niger, Sudan, Tanzania, Zimbabwe, Zambia, Uganda, Cameroon, Ghana	Paediatric surgical conditions
Sanz Cortes et al. (2021) [42]	International Fetoscopic Neural Tube Defect Repair Consortium	South Africa	Fetoscopic surgery Paediatric surgery
Biccard et al. (2020) [43]	APORG, African Peri-operative Research Group working group	South Africa, Uganda, Nigeria, Ghana, Botswana, Kenya, Mozambique, Tanzania, Ivory Coast, Ethiopia, Eswatini, Namibia, Sierra Leone, Botswana, Liberia, Egypt, Zambia, Senegal, Congo, Madagascar, Mali, Benin	Perioperative research
Brenner et al. (2020) [44]	CRASH-3 trial collaborators, corticosteroid randomization after significant head injury	Cameroon, Nigeria, Kenya, Zambia,	Improving outcome of traumatic brain injury Neurosurgery, trauma surgery
Chu et al. (2020) [45]	AfroSurg Collaborative	South Africa	Access to safe surgery in Africa Health system management
Delisle et al. (2020) [46]	SOS & GlobalSurg, Surgical Outcomes Study Groups and GlobalSurg Collaborative	Algeria, Benin, Burundi, Cameroon, Congo, DRC, Egypt, Ethiopia, Gambia, Ghana, Kenya, Libya, Madagascar, Mali, Mauritius, Namibia, Niger, Nigeria, South Africa, Tanzania, Togo, Uganda, Zambia, Zimbabwe	Access to safe surgery in Africa General surgery, anaesthesia, health system management
Edlmann et al. (2020) [47]	iCORIC, International Collaborative Research Initiative on Chronic Subdural Haematoma study group	Ethiopia	Chronic subdural hematoma Neurosurgery
EuroSurg et al. (2020) [48]	EuroSurg Collaborative	South Africa	International surgical clinical audits General surgery, colorectal surgery
Ford et al. (2020) [49]	OxPLORE Collaboration, Oxford Pediatrics Linking Oncology Research with Electives	Tanzania, Rwanda	Student-led research collaboration; Wilms’ tumour Paediatric surgery
GlobalSurg et al. (2020) [50]	GlobalSurg Collaborative	Egypt, Ethiopia, Ghana, Nigeria, Sierra Leone, South Africa, Zambia, Burundi, Botswana, Malawi, Morocco, Rwanda, Kenya	Surgical site infections in children Paediatric surgery
Held et al. (2020) [51]	LION, Learning Innovation via Orthopaedic Networks Group	South Africa, Ghana, Malawi, Tanzania, Kenya, Namibia	Orthopaedic training in Southern Africa
Lynch et al. (2020) [52]	ARGO, African Research Group for Oncology Collaborative	Nigeria	Oncology research, breast biopsy Oncology
Muhly et al. (2020) [53]	PPOG, Pediatric Perioperative Outcomes Group	South Africa	Paediatric perioperative Outcomes Perioperative care
Pindyck et al. (2020) [54]	African Intussusception Surveillance Network	Ethiopia, Ghana, Kenya, Malawi, Tanzania, Zambia, Zimbabwe	Intussusception Paediatric surgery
Protopapas et al. (2020) [55]	COVID-19 International Congenital Heart Surgery Taskforce	Ghana, South Africa	Impact of COVID-19 on paediatric cardiac surgery
Robertson et al. (2020) [56]	WFNS Young Neurosurgeons Committee, World Federation of Neurosurgical Societies Young Neurosurgeons Committee	Rwanda, Morocco, Algeria, Ethiopia, Cameroon	Neurosurgery training
McMeekin et al. (2020) [57]	OVIVA collaborators, Oral versus Intravenous Antibiotics for Bone and Joint Infection	Kenya	Antibiotics for orthopaedics infections
Robertson et al. (2020) [58]	Collaborative Working Group	Cameroon, Malawi, South Africa, Rwanda	Task shifting and sharing in neurosurgery in LMIC
Papadopoulos et al. (2020) [59]	CGRN, International Study of Childhood Glaucoma — Childhood Glaucoma Research Network Study Group	Ghana	Childhood glaucoma Ophthalmology
Brenner et al. (2019) [60]	HALT-IT Trial Collaborators, Haemorrhage Alleviation with Tranexamic acid-Intestinal system	Nigeria, Egypt, Sudan	RCT on GIT bleeding and tranexamic acid General surgery
A. E.-A. H. A. R. C. S. M. group et al. (2019) [61]	E-AHPBA, European-African HepatoPancreatoBiliary Association Research Collaborative Study management group	South Africa	Hepatobiliary disease General surgery, hepatobiliary surgery
Dewan et al. (2019) [62]	PGSS, Program in Global Surgery and Social Change collaborators	South Africa, Uganda	Access to safe neurosurgery in LMCI
GlobalSurg (2019) [63]	GlobalSurg Collaborative	Benin, Cameroon, Egypt, Ethiopia, Ghana, Libya, Malawi, Mozambique, Nigeria, Rwanda, South Africa, Tanzania, Zambia, Sudan	Colostomy and colorectal resection General surgery
GlobalSurg (2019) [64]	GlobalSurg Collaborative	Benin, Botswana, Burundi, Cameroon, Egypt, Ethiopia, Ghana, Libya, Kenya, Madagascar, Morocco, Malawi, Mozambique, Nigeria, Rwanda, South Africa, Sudan, Tanzania, Zambia	WHO Surgical Safety Checklist use audit General surgery
Iverson et al. (2019) [65]	Safe Surgery 2020 Collaborators	Ethiopia	Access surgical capacity Health system management
Li et al. (2019) [66]	OVIVA Trial Collaborators, Oral versus Intravenous Antibiotics for Bone and Joint Infection	Kenya	Antibiotics for orthopaedic infections
Robertson et al. (2019) [67]	Global Neurosurgery Survey Collaborators	Cameroon, Malawi, South Africa, Rwanda	Task shifting and sharing in neurosurgery
Fink et al. (2018) [68]	Pediatric Acute Lung Injury and Sepsis Investigators (PALISI) Network, PALISI Global Health Subgroup, and Prevalence of Acute Critical Neurological Disease in Children: A Global Epidemiological Assessment (PANGEA) Investigators Pediatric Acute Lung Injury and Sepsis Investigators, Prevalence of Acute Critical Neurological Disease in Children: A Global Epidemiological Assessment Investigators	Kenya, Ethiopia, Rwanda, Ghana	Epidemiology and outcome of TBI & infectious encephalopathy Paediatric Surgery, Neurosurgery
GlobalSurg (2018) [69]	GlobalSurg Collaborative	Benin, Botswana, Burundi, Egypt, Ethiopia, Ghana, Madagascar, Malawi, Nigeria, South Africa, Sudan, Zambia	Surgical site infection after GI surgery General surgery
Goodman et al. (2018) [70]	GICS Collaborators, Global Initiative for Children’s Surgery	Nigeria	Optimizing children’s surgical care in LMIC Paediatric surgery
Israels et al. (2018) [71]	The Collaborative Wilms Tumour Africa team	Malawi, Cameroon, Ghana, Zimbabwe, Uganda, Ethiopia	Wilms’ tumour Paediatric surgery
Tate et al. (2018) [72]	African Intussusception Surveillance Network	Conga, Ethiopia, Ghana, Kenya, Malawi, Tanzania, Zambia, Zimbabwe	Intussusception Paediatric surgery
Reilingh et al. 2018 [73]	International Consensus Group on Cartilage Repair of the Ankle	South Africa	Ankle repair surgery Orthopaedics
Van Dijk et al. (2018) [74]	International Consensus Group on Cartilage Repair of the Ankle	South Africa	Ankle repair surgery Orthopaedics
Sprague et al. (2018) [75]	INORMUS Investigators, International Orthopaedic Multicentre Study	Uganda, Kenya, Tanzania, South Africa, Nigeria, Botswana, Ghana	Musculoskeletal trauma in LMCIs Orthopaedics
Dresser et al. (2017) [76]	Global Emergency Care Collaborative Investigators	Uganda	Emergency surgical care
Brink et al. (2017) [77]	NASSA, Netcare Antimicrobial Stewardship Study Alliance	South Africa	Peri-operative antibiotic use
Ekure et al. (2017) [78]	Nigerian Pediatric Cardiology Study Group	Nigeria	Paediatric heart disease/congenital heart defects
Czauderna et al. (2016) [79]	CHIC, The Children’s Hepatic Tumors International Collaboration	Malawi	Children hepatic tumours
Yang et al. (2016) [80]	Africa Network for Gastrointestinal and Liver Diseases	Cameroon, Egypt, Ethiopia, Ghana, Ivory Coast, Nigeria, Sudan, Tanzania, Uganda	Hepatocellular carcinoma in Africa General surgery
Mwinga et al. (2015) [81]	SIRCLE Collaboration	Kenya	Quality of surgical care Health system management

### Year of publication and authors

A majority of 33/55 (60%) of the studies included were published in 2020 (17 papers) and 2021 (16 papers). None of the studies included were published prior to 2015. However, 14/55 (25%) studies began data collection between 2011 and 2015, but the articles were completed and published later. Only 4/55 (7%) of the included studies started prior to the year 2011, with the majority 12/55 (21%) starting after 2019.

A review of the authors found that 8/44 (18%) had published more than one paper during the 10-year period reviewed. GlobalSurg [50, 63, 64, 69] published the highest number (4), followed by Robertson et al. [56, 58, 67], with three articles (Table 2), while only 6/55 (11%) of the studies had an African-based first author in the publication.

**Table 2:** List of authors with more than one study included in the review

Author(s)	Number of studies
GlobalSurg	4
Robertson et al	3
Brenner et al	2
AfroSurg Collaborative	2
Oral versus Intravenous Antibiotics for Bone and Joint Infection	2
National Institute for Health Research	2
African Intussusception Surveillance Network	2
International Consensus Group on Cartilage Repair of the Ankle	2

### Study designs

Ten main study methods were used throughout the included studies (Supplementary Table 4). The prospective, observational cohort study was the most frequently used methodology and represented 14/55 (25%) of the articles reviewed. This was followed by Delphi and Expert opinion studies, representing 10/55 (18%) of the articles. Finally, cross-sectional survey and retrospective, observational study design, each accounted for 8/55 (15%) of the publications.

### Continents and countries

**Fig. 2:**
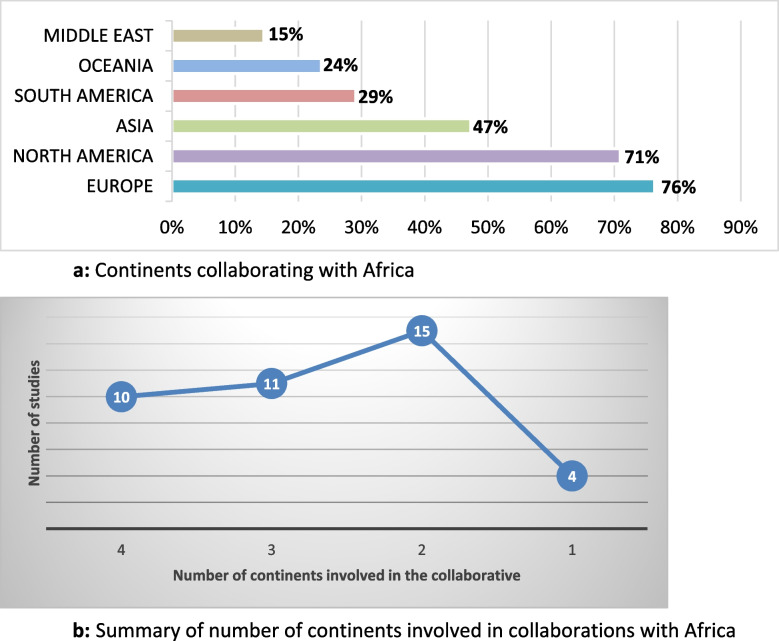
**a** Continents collaborating with Africa. **b** Summary of number of continents involved in collaborations with Africa

The collaborations included all continents. It was noted that Africa mainly collaborated with Europe, i.e. 42/55 (76%) studies (Fig. 2a), followed by North America in 39/55 (71%) studies, and the least with the Middle East in 8/55 (15%) studies. A minority of the studies 4/55 (7%) were conducted only between African countries (Fig. 2b).

A total of 34 (63%) out of 54 African countries participated in the collaborative studies selected. Of these, 29/54 (54%) were in sub-Saharan Africa. The different African countries participated a total of 266 times in the 55 studies selected (Supplementary Table 5).

**Fig. 3:**
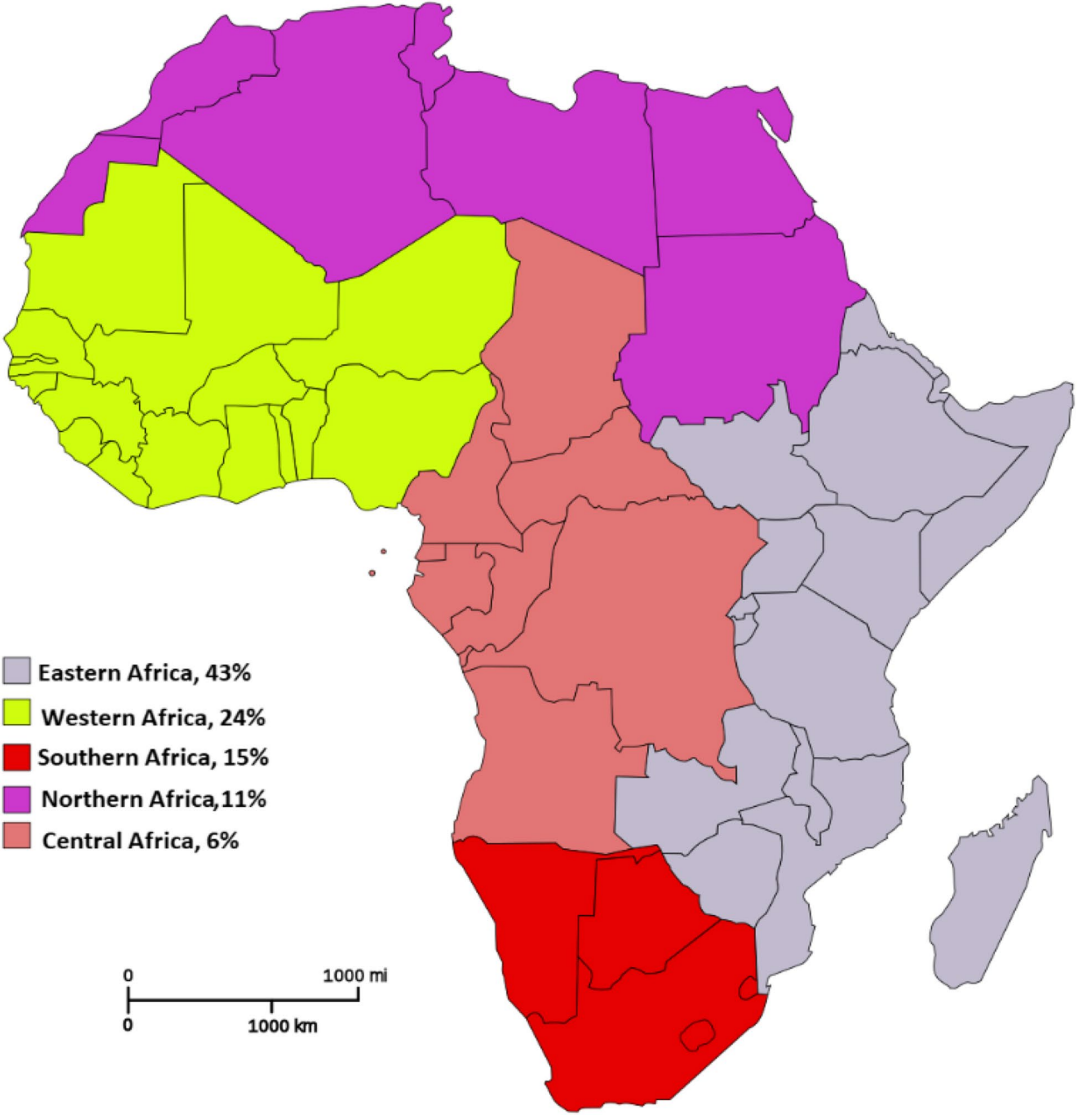
Collaborative surgical publications by region in Africa (adapted from Wikipedia: Common Africa Map [82])

The majority of the countries involved were from Eastern Africa 114/266 (43%), followed by West Africa 65/266 (24%), Southern Africa, 40/266 (15%), North Africa 30/266 (11%), and Central Africa which had the least 17/266 (6%) (Fig. 3). The countries with the highest volume of publications were South Africa 29/266 (11%), Nigeria 21/266 (8%), Ethiopia 19/266 (7%), and Kenya 18/266 (7%).

### Number of centres/registries and collaborators

The studies included multiple centres, hospitals, and registries. On average, each study included 93 centres. The highest number from a single study was 1464 centres, and the minimum was a single centre. On average, each study had 382 collaborating investigators. The highest number of collaborators in a single study was 3445, and the lowest was only six.

### Surgical specialties

The papers were categorized into a total of 14 surgical specialty fields (Supplementary Table 6). Some of the studies involved multiple specialties. The top surgical specialties were as follows: neurosurgery 14/84 (17%), paediatric surgery 14/84 (17%), general surgery 12/84 (14%), and orthopaedic surgery 10/84 (12%). The surgical specialty with the lowest volume of collaborative surgical research was upper gastrointestinal (GI) 1/84 (1%).

### Facilitating factors and challenges

The review explored and identified the factors that facilitate the collaborations as well as challenges experienced. We directly extracted explicit statements from the publications relating to these factors. The factors were then grouped into six thematic categories for ease of analysis: (i) structure and design, (ii) information and communication, (iii) resources, (iv) networks, (v) ethics, and (v) other (Supplementary Table 2). Approximately, 22/55 (40%) of the articles [29, 34, 44–47, 51–54, 57, 60–62, 66, 68, 70, 72–74, 76, 80] did not describe their facilitating factors, and neither did 18/55 (33%) studies [29, 32, 42, 44, 47, 50, 51, 53–55, 57, 62, 66, 72–74, 76, 81] document challenges limiting the collaborations. A complete list of the facilitating factors and limitations is summarized in Supplementary Table 7 below.

#### Structure and design

A clear structure with terms of reference, a central management team, multidisciplinary groups, a division into smaller teams with experienced leaders, and goal and target setting could facilitate these collaborations [39, 48, 50, 63, 64, 69, 75, 79, 81]. Problems included poor recruitment of local investigators, especially in LMICs and Africa, dropout of investigators across multiple rounds of the same study, power imbalances between HIC and LMIC authors, a skewed data representation of the identified regions, variations in the local and regional context, and difficulties in achieving consensus [27, 30, 31, 33, 35, 36, 39, 40, 43, 49, 63, 67, 75, 80].

#### Information and communication

Information and communication included maximizing the initial recruitment of collaborators into the study using multiple channels such as social media, videos, personal emails, and promotions. Online communication tools, electronic data capture, and information systems greatly facilitated the proliferation of collaboratives [27, 28, 30, 32, 33, 35–43, 48, 50, 55, 56, 58, 59, 67, 69, 81]. These were shown to be more successful when augmented with physical and offline modes of communication, such as face-to-face meetings. Unfortunately, participants in regions with poor access to the Internet were at risk of being left out of these studies [37, 39, 56, 58]. Collaborative studies were performed across a wide range of regions, and the use of multiple languages was found to facilitate these studies. However, this introduced a new challenge as the collaboratives needed to have the capacity and resources to not only translate the language but also understand the cultural and social context of the different regions the studies were conducted [28, 65, 67].

#### Resources

It was observed that access to adequate funding and other resources, such as human capital, facilitated collaboration. HICs had higher access to these resources and utilized this advantage to partner with collaborators in Africa and other LMICs [28, 39, 43, 49, 69, 75, 81]. However, African researchers had lower access to financial resources, limited research support from their home institutions and countries, and a limited number of experts and specialists [27, 28, 31, 33, 45, 49, 65, 78, 80].

#### Networks

The use of networks from known groups, associations, or personal and professional relationships was seen to facilitate collaboration [27, 28, 41, 49, 56, 58]. However, this was a challenge when the collaborators did not have such networks in the regions where they planned to conduct their studies or if they needed to travel to establish these relationships [31, 35, 52, 80].

#### Ethics

Ethical approval was an advantage when the multicentre study was conducted in a single region and country. This was due to the easy application of a single approval process across multiple research sites [36, 39, 43]. However, it proved challenging when the studies required multiple ethical approvals from each institution or region where the collaborators were located. In addition, the research studies needed adequate planning, funding, and resources to be successful in such situations. This was seen to be a significant limitation, especially in LMICs [48, 60, 61, 68, 69].

## Discussion

### Trends in collaborative publications

There has been a general increase in the global volume of international collaborative research. The recent COVID-19 pandemic further highlighted the benefit of international research collaboration [1, 83]. Kim et al. (2020) described an increase in multinational co-authored papers from a low of approximately 10% in the 1990s to a high of 25% by 2018 [84]. Some studies additionally found that the pandemic led to an increase in the number of international research collaborations. However, we could not find literature that has quantified the current rate of international collaborative studies [83, 85]. Kim et al. (2020) also observed that the USA, China, and the UK had the highest contribution to global surgical research, while LMICs had the lowest contribution rates [83]. However, Honeyman et al. (2021) note that the pandemic led to a decline in international surgical collaborations, thus further limiting access to surgical care in LMICs [1]. Weiner et al. (2020) similarly noted a disruption and decline in research unrelated to COVID-19 [86]. Lee et al. (2021) described that despite an overall increase in collaborative research during the COVID-19 pandemic, some countries experienced an overall decline in collaborations. However, it was observed that LMICs and countries significantly impacted by the pandemic had an increase in collaborative research specifically related to COVID-19 [85].

Our research found increasing participation in collaborative studies in Africa. Seventy-five percent of the studies reviewed started and were published from 2019 to 2021. This is supported by Nepogodiev et al. (2017), who described a rising trend in international collaborations in HICs [22]. Facilitated by established infrastructure and administration, research funding, and training support, trainee-led surgical collaboratives have grown to involve 99% of UK Hospitals over 10 years [22]. However, literature describing the rate of increase in collaborative studies in Africa could not be found.

### Study designs

Prospective observational cohort studies accounted for a majority (25%) of the collaborative research publications we identified. Randomized control trials contributed to only 5% of these papers. Huamaní et al. (2013) similarly reported that a minority (< 20%) of surgical clinical trials performed between 2007 and 2013 were conducted by international research collaboratives. Additionally, they found that international collaborations were more common in nonsurgical fields [13]. We did not explore nonsurgical international collaborative studies.

### Continents and countries

Kim et al. (2020) found that the largest volume of research globally was from the USA, China, and the UK in order [83]. However, we observed a low collaboration volume between Africa and Asia, despite China producing high volumes of collaborative research globally in recent years. Kim et al. (2020) additionally observed that despite the close proximity, Asian countries rarely collaborated, while the USA and Europe actively collaborated. They proposed the possibility of having different research interests as a contributing factor [83]. Analogous factors might affect the degree of collaboration between African countries that are also close. There is a need to understand the relationship and geopolitical factors that could affect research collaboration between African countries and Asia.

Confraria et al. described “colonial ties” in research collaborations by observing that African countries tended to collaborate more with the nations that colonized them compared with other countries [87]. The lowest collaborations occurred intra-Africa and between Africa and the Middle East. This can be a consequence of the many common limiting factors across LMICs, such as lack of funding and low number of researchers.

A report of a 10-year collaboration between two African countries (Mozambique and Uganda) by Namuyonga et al. (2018) found that collaboration between LMICs in Africa can be feasible to promote high-quality research and improved clinical skills through community-based approaches, research mentorship, and hands-on skills training that is often not available to African counterparts in North–South collaborations [20]. A critical facilitating factor for these collaborations was access to funding, which enabled access to translation services, therefore overcoming language barriers [20].

### Facilitating factors and challenges

#### Structure and design

Fischer et al. [18] described a step-by-step process to establishing a successful collaboration. Although not in chronological order, our study found similar facilitating factors. Our review showed that researchers from Africa and other LMICs often lack the financial resources to travel for international conferences. Therefore, unless multiple recruitment channels are explored, researchers from Africa and other LMICs are at risk of being underrepresented or completely excluded. We found that collaborators frequently had difficulty in achieving consensus between members This was further complicated by power imbalances between LMICs and HICs that have easier access and often control the financial resources. In general, we observed that contributors from Africa had lower representation and acknowledgment, especially as first or lead authors.

#### Information and communication

There is an opportunity for surgeons and researchers in the global south to drive global surgery and collaboration through leveraging of information technology such as social media use. Navarro et al. (2020) observed that despite high mobile phone penetration and high social media usage in Africa and other LMICs, these countries produced low volumes of global surgery content on social media [9]. They additionally noted that only 20% of the social media users accounted for approximately 70% of the surgical content generated; they were termed social media influencers. These influencers were found to have a wide-reaching audience. Therefore, if positioned correctly, even with the low number of experts in Africa, the few can still have a huge impact compared to traditional platforms [9].

IT platforms, however, are hindered by the poor access to the Internet and technology in Africa [14]. This limits participation in virtual conferences and the use of other IT tools that have been premised to have the capacity to help LMICs leapfrog to modern collaborative and global surgical research methodology. Sonshine et al. (2013), in a study on collaboration in Nigeria, found that Facebook provided a more reliable form of communication, document sharing, and collaboration as compared to emails [88]. Ethical concerns and other risks must be considered versus the perceived benefits before large-scale adoption of new solutions.

#### Resources

Access to funding has been cited as an essential facilitating element. Adequate funding is crucial, since it allows the researchers to focus on actual research rather than administrative work, which may be “outsourced” [18]. Sonshine et al. (2013) hypothesized that financial barriers were the most significant hindrance to successful research collaborations in LMICs. They further noted that until financial and resource-based barriers were addressed, other challenges such as administrative, technological, and human resource factors would not be resolved [88].

Researchers from Africa and other LMICs are indirectly limited from accessing financing due to a lack of research support and management capacity. Fischer et al. (2017) highlights the need for training in research management, administration, and grant writing. They reported that such skills and capabilities were often missing in LMICs [18]. Our study similarly outlined this as a barrier cutting across many LMICs and African countries.

#### Networking

The networking power of an African doing a postdoctoral degree (such as a Masters or PhD) abroad has also been shown to be facilitating factor in both initiating and maintaining long-term research partnerships [87]. Over 80% of global health research organizations are located in HICs; therefore, most international conferences are held in these regions. The financial barriers and visa restrictions make it difficult for individuals from LMICs to attend these events. It has been demonstrated that for each 10% increase in US visa rejection rates for a country, there is a commensurate 23% decline in conference speaker attendees from the country [10]. The shift to virtual conferencing during COVID-19 has assisted in reducing conference equity; however, social exchange, networking, and collaboration are not as strong from online events compared to physical events [10, 89].

#### Ethics

International collaborative research can be limited by the different ethical requirements needed to conduct research in various regions. Therefore, collaborative researchers need to plan for the different ethical requirements and processes when preparing for their studies [90]. Currently, no formal guidelines exist to guide different stakeholders to navigate ethical challenges in collaborations with LMICs, especially so in global surgery collaborations [84, 91]. Challenges such as the need for “double ethics” from both the host and visitor institutions or countries are commonly described in literature [91]. It has been demonstrated that ethical guidelines from developed countries are not directly applicable to developing countries due to different social, cultural, and religious contexts [84]. International research must be impactful to local communities and mutually beneficial to all stakeholders [91]. This is even further undermined by the practice of “ethical dumping” defined by the European Commission in 2016 as the practice where HIC researchers choose to undertake a study that does not meet ethical requirements in their home country in LMICs to take advantage of poorer ethical approval systems and structures [92, 93].

#### Contribution and recognition

Ravi et al. (2021) observed that historically, LMICs are generally marginalized from global surgical research [14]. African-based researchers are more likely to participate as data collectors and rarely appear as lead authors in international research studies [87]. This issue disproportionately, and negatively affects women in LMICs who receive the least representation compared to female authors in HICs. They found systemic biases against female academics participating in collaborations in HIC-LMIC partnerships [14]. In general, HICs have been shown to have higher authorship appearance in publications due to a better organized research infrastructure demonstrated by the ease of access to academic resources, uniformity in language with minimal translation needs, access to protected research time, and availability of higher investment in research and access to resources through grants and related funding opportunities [14].

Collaboratives should have an open and deliberate discussion based on contributions made to determine the authorship in accordance with the International Committee of Medical Journal Editors (ICMJE) ethics and guidelines, so as to provide equal opportunity for listing LMIC researchers as first and senior authors. This will help eliminate the dissatisfaction from under recognition of these authors, which may trigger conflict between the consortia members. Each collaborator should be equitably recognized for their contribution [18].

### Limitations

The study covered a 10-year period, and only reviewed two indexed databases. A longer time frame and a wider search strategy would have been employed to gather more data and view trends across different periods including specific changes that may be related to isolated disease outbreaks (e.g. COVID-19) and the impact that these had on the uptake of collaborative research. As stated in the methodology, the study did not examine all surgical disciplines excluding obstetrics and gynecology and dental surgery. The search may have missed out on surgical specialties that do not typically use the term surgery, e.g. burns. However, our selection was based on the feasible time and resources available to conduct the study. We excluded studies that were not published in English, which could have biased the results; however, lack of translation capacity necessitated this. Information on facilitating factors and challenges were not explicitly expressed in all the articles reviewed and thus relied on the subjective interpretation of the reviewers. Thirty percent (16/55) of the articles did not describe their facilitating factors, nor did 40% (22/55) of the studies document challenges limiting the collaborative study design. Furthermore, some factors, such as ethics, were implicitly stated and would benefit from an in-depth survey or interview with the collaborators.

In addition, due to resource limitations, time constraints, and anticipated delays in receiving ethical approvals, we did not pursue the use of interviews or other data collection methods to uncover more information that was not available in the publications [94].

## Conclusion

We found a low volume of international research collaboration with Africa compared to HICs, although the number of studies and collaborations in the region is increasing. We conducted a retrospective review; however, a detailed prospective study on ongoing collaborations is needed to further explore, per region, the different issues presented in this paper which would help provide a deeper understanding that would inform long-term solutions that encourage collaborative studies in Africa and other geographies. This may need to be a different study design, possibly qualitative with narratives.

### Supplementary Information

**Additional file 1: Supplementary Table 1.** Search terms. **Supplementary Table 2.** Facilitating factors and challenges extracted for each collaboration. **Supplementary Table 3.** Studies included in systematic review of surgical collaboratives. **Supplementary Table 4.** Summary of study designs. **Supplementary Table 5.** Summary of participation in collaborative surgical publications in Africa (by region). **Supplementary Table 6.** Statistics of the surgical specialties from the studies reviewed. **Supplementary Table 7.** List of Facilitating factors & challenges affecting collaborative research studies. **Supplementary Table 7a.** List of Facilitating Factors affecting surgical collaboratives. **Supplementary Table 7b.** List of challenges to surgical collaboratives.

## Data Availability

The datasets generated and/or analysed during the current study are available in the searchRxiv, CABI International, Repository, 10.1079/searchRxiv.2023.00235, 10.1079/searchRxiv.2023.00236 [25, 26].

## Abbreviations

COVID-19: Coronavirus disease 2019
LMICs: Low- and middle-income countries
GI: Gastrointestinal
HICs: High-income countries
ICMJE: International Committee of Medical Journal Editors
IT: Information technology
KEMRI: Kenya Medical Research Institute
LMICs: Low- and middle-income countries
MeSH: Medical Subject Headings
NIHR: National Institute for Health Research
PhD: Doctor of Philosophy degree
PRISMA: Preferred Reporting Items for Systematic Reviews and Meta-Analyses
RCT: Randomized control study
REDCap™: Research Electronic Data Capture
SARS-CoV-2: Severe Acute Respiratory Syndrome caused by Coronavirus (type 2)
SDGs: Sustainable Development Goals
sSA: Sub-Saharan Africa
UK: United Kingdom
UN: United Nations
USA: United States of America
