# Supplementary material for: Catalysing global surgery: a meta-research study on factors affecting surgical research collaborations with Africa
Source: Syst Rev. 2024 Mar 18;13:89. doi: 10.1186/s13643-024-02474-8 (PMC10946148; doi:10.1186/s13643-024-02474-8)
Supplement: Supplementary file 1 — Additional file 1: Supplementary Table 1. Search terms. Supplementary Table 2. Facilitating factors and challenges extracted for each collaboration. Supplementary Table 3. Studies included in systematic review of surgical collaboratives. Supplementary Table 4. Summary of study designs. Supplementary Table 5. Summary of participation in collaborative surgical publications in Africa (by region). Supplementary Table 6. Statistics of the surgical specialties from the studies reviewed. Supplementary Table 7. List of Facilitating factors & challenges affecting collaborative research studies. Supplementary Table 7a. List of Facilitating Factors affecting surgical collaboratives. Supplementary Table 7b. List of challenges to surgical collaboratives. [file 13643_2024_2474_MOESM1_ESM.docx]

## Supplementary Table 1 - Search terms

| **Surgery** | **Collaborative** | **Region- Africa** | **10-year period** |
| --- | --- | --- | --- |
| Surg* or “global surg*” | Collab* or consort* or coalition or network or alliance or forum or platform | Africa or “Africa south of the Sahara" or “sub-Saharan Africa” or Angola or Benin or Botswana or Burkina Faso or Burundi or Cameroon or Cape Verde or Central African Republic or Chad or Comoros or Congo or Cote d'ivoire or Djibouti or Equatorial Guinea or Eritrea or Ethiopia or Gabon or The Gambia or Ghana or Guinea or Guinea-Bissau or Kenya or Lesotho or Liberia or Madagascar or Malawi or Mali or Mauritania or Mauritius or Mozambique or Namibia or Niger or Nigeria or Reunion or Rwanda or "Sao Tome and Principe" or Senegal or Seychelles or Sierra Leone or Somalia or South Africa or Sudan or Swaziland or Tanzania or Togo or Uganda or Western Sahara or Zambia or Zimbabwe | 1^st^ January 2011 to 31^st^ September 2021 |
| The search included Surg*, collab*, and consort* which were chosen as stem words for surgery, collaborative, collaboration, and consortium. | | | |

## Supplementary Table 2: Facilitating factors and challenges extracted for each collaboration

| **Category** | **Example Facilitating factors** | **Example Challenges** |
| --- | --- | --- |
| **1. Information & communication** | Use of online communication | Use of multiple languages |
| **2. Structure & Design** | Clear structure & terms of reference | Difficulty in achieving desired diversity of collaborators |
|  | Setting clear goals & targets | Difficulty in achieving consensus between members |
| **3. Resources** | Secure and adequate financial resources | Limited access to resources |
|  | Running smaller pilot studies to optimize study design | Poor research support for investigators |
| **4. Ethics** | Single ethical approval for multicentre studies | Multiple ethical approvals resulting in increased costs, and delays |
|  | Identifying ethical requirements for different regions |  |
| **5. Network** | Use of existing networks and relationships | Need for personal networks and relationships to improve recruitment |
|  | Support from associations, groups, and networks |  |
| **6. Other** |  | COVID-19 disruption to funding and healthcare delivery |

**Supplementary Table 3:** Studies included in systematic review of surgical collaboratives

| **Author, & publication year** | **Study method** | **Year of research** | **Acronym &**  **Name of collaborative^*^** | **Continent(s)** | **African country(s) involved** | **Size/ number of hospitals, centres, & registries** | **Number of collaborators** | **Surgical specialty** | **Research scope/ focus** | **Facilitating factors** | **Challenges** |
| --- | --- | --- | --- | --- | --- | --- | --- | --- | --- | --- | --- |
| Abbas et al, 2021, (24) | Expert opinion, Online Action Planning Forum | 2020 | GICS,  [Global Initiative for Children’s Surgery](https://pubmed.ncbi.nlm.nih.gov/?term=Global+Initiative+for+Children%E2%80%99s+Surgery%5BCorporate+Author%5D) | Asia, North America, Africa, South America, Europe | Nigeria, Kenya, Egypt | 47 | 16 | Paediatric surgery | Impact of COVID-19 on Global Paediatric surgery | 1. Existing network in both LMICs & HICs  2. Use of online resources:  a. action planning forum  b. sharing of information on their website  c. email  3. Use of Zoom platform sponsored by Lifebox  4. Cost reduction:  a. Pre-registration at no cost to participants  b. No travel costs  5. Online forums held at 1400-1530 Greenwich Meridian Time to facilitate participation from different time zones  6. Majority of panelists were from LMICs thus improving participation  7. Easily replicable methodology | 1. Limited resources in LMICs  2. Drop in participation between 1^st^ and 2^nd^ online forum  3. Webinar fatigue  4. Availability of forum discussions later in recorded videos, YouTube^TM^, and articles. This discourages live attendance & participation needed for collaboration, as participants are able to receive discussion proceedings at a later date.  5. Proposed solutions/ actions were beyond the scope of the study group  6. Need to assess impact of the solutions developed which may require a different study methodology |
| Alkhaffaf et al, 2021, (25) | Systematic review, Cross-sectional survey, Delphi study, Case study | 2019 | [GASTROS International Working Group](https://pubmed.ncbi.nlm.nih.gov/?term=GASTROS+International+Working+Group%5BCorporate+Author%5D),  Gastric Cancer Surgery Trials Reported Outcome Standardization | Africa, Asia, South America, Europe | Nigeria | 11 | 28 | General Surgery | Gastric Cancer Surgery | 1. Use of local language  2. Standardized Delphi surveys  3. Clear promotion of the study and its aims to the relevant stakeholders  4. Ensure local perspectives are represented & preserved  5. Using both internet and paper-based platforms for those without access to internet  6. Maximizing recruitment through: social media use, multimedia (such as videos), personal emails, personal networks, defining a recruitment strategy, & dissemination strategy  7. Identifying different ethical approvals needed per region  8. Early financial planning and appropriate resource allocation  9. Employing professionals to undertake some aspects of the study e.g., translations  10. Collaborators signed terms of reference document with outlined benefits, & responsibilities.  11. Regular communication with collaborators  12. Clear goal/ target setting e.g., number of surveys per collaborator  13. Support from influential stakeholder groups/ associations and networks  14. Use of IT platforms to manage the studies | 1. Translation/ harmonization across multiple languages  2. Conducting multiple stage/ round studies increased the time taken  3. Multiple ethical approvals needed. Cost and time factors.  4. Cost of Information Technology (IT) platforms  5. Need for financial resources  6. No standardized approach to this field |
| Alphonsus et al, 2021 (26) | Prospective, observational cohort | 2019-2020 | [Collaborators for EPIC2: BNP study](https://pubmed.ncbi.nlm.nih.gov/?term=Collaborators+for+EPIC2%3A+BNP+study%5BCorporate+Author%5D) | Africa | South Africa | 7 | 42 | Perioperative care | Cardiovascular risk stratification in surgery | Not described | Not described |
| Beattie et al, 2021 (27) | Delphi study, Systemic review | 2017 | [StEP COMPAC Group](https://pubmed.ncbi.nlm.nih.gov/?term=StEP+COMPAC+Group%5BCorporate+Author%5D),  the Standardized Endpoints in Perioperative Medicine Core Outcome Measures in Perioperative and Anesthetic Care | North America, Africa, Europe, Australia | South Africa | N/A | 122 | Perioperative care | Perioperative cardiovascular adverse events | 1. Communication: use of reminder emails to encourage participation | 1. Time constraints with multiple rounds of Delphi surveys  2. Difficulty in achieving consensus between the members |
| Breedt et al, 2021 (28) | Expert opinion | 2020 | [AfroSurg Collaborative](https://pubmed.ncbi.nlm.nih.gov/?term=AfroSurg+Collaborative%5BCorporate+Author%5D) | Africa, North America, Europe, | South Africa, Botswana, Namibia | N/A | 29 | Health system management | Surgical Safety | 1. Orientation of participants to research methodology  2. Collaborators with a wide range of backgrounds & specialties | 1. Lack of experience of participants with research methodology  2. Lack of lead authors from LMICs  3. Need to secure funding  4. Poor information systems  5. Communication: language/ cultural barriers, limited communication platforms, need for physical meetings  6. Small number of collaborators |
| Firth et al, 2021 (29) | Retrospective, observational | 2019 | [Mbarara SQUAD Consortium](https://pubmed.ncbi.nlm.nih.gov/?term=Mbarara+SQUAD+Consortium%5BCorporate+Author%5D),  Surgical Quality Assurance Database | Africa, North America | Uganda | 1 | 31 | General surgery, Trauma, Perioperative, Anaesthesia | Surgical Safety | 1. Information systems: availability of a hospital medical record system & database  2. Single centre ethical approval | Not described |
| Held et al, 2021 (30) | Delphi study | 2019-2020 | [Authorship group ‘Knee surgery in LRS’](https://pubmed.ncbi.nlm.nih.gov/?term=Authorship+group+%E2%80%98Knee+surgery+in+LRS%E2%80%99%5BCorporate+Author%5D) | Africa, Europe, Asia, North America, South America | South Africa | 6 | 41 | Orthopaedics | Knee surgery | 1. Use of emails with weekly reminders  2. Electronic data collection using Research Electronic Data Capture (REDCap^TM^) | 1. Difficulty finding specialist providers in areas with limited resources  2. A higher number of participants from high income countries  3. Difficulty in achieving consensus |
| Kachapila et al, 2021 (31) | Modelling study, prospective cohort, expert opinion | 2020 | NIHR Global Health Research Unit on Global Surgery; ASOS Investigators; STAR Surg Collaborative,  National Institute for Health Research | Africa, Europe, Oceania | Algeria, Benin, Burundi, Cameroon, Congo, DRC, Egypt, Ethiopia, Gambia, Ghana, Kenya, Libya, Madagascar, Mali, Mauritius, Namibia, Niger, Nigeria, Senegal, South Africa, Tanzania, Togo, Uganda, Zambia, Zimbabwe | N/A | 3415 | Health system management | Lowering surgery hospital costs | Not described | 1. Using cost data from different countries |
| Kanmounye et al, 2021 (32) | Cross-sectional study | 2018 | [CAANS Young Neurosurgeons Committee and WFNS Young Neurosurgeons Committee](https://pubmed.ncbi.nlm.nih.gov/?term=CAANS+Young+Neurosurgeons+Committee+and+WFNS+Young+Neurosurgeons+Committee%5BCorporate+Author%5D),  Continental Association of African Neurosurgical Societies & The World Federation of Neurosurgical Societies | Africa, North America, Asia, Europe | Cameroon, South Africa, Liberia, Côte d'Ivoire, Nigeria, Zimbabwe, Senegal, Egypt, Kenya, Rwanda, Morocco, Algeria, Ethiopia, Niger, Congo, Uganda | 20 | 30 | Neurosurgery | Neurosurgical training in Africa | 1. Use of personal emails and social media platforms (Twitter, Facebook, and WhatsApp) | 1. Collaborators with poor access to reliable internet and electronic devices are missed out  2. Low exposure to research for collaborators from Africa i.e., institutions don’t have journal clubs, lack of assigned/ protected research time |
| Masters et al, 2021 (33) | Prospective, observational cohort | 2019 | ORCA,  [Orthopaedic Research Collaboration for Africa Orca Investigators](https://pubmed.ncbi.nlm.nih.gov/?term=Collaboration+For+Africa+Orca+Investigators+OBOTOR&cauthor_id=34382549) | Africa | South Africa | 52 | 10 | Orthopaedics | Gunshot related Orthopaedic trauma | 1. Use of a secure online information system to capture study data i.e., REDCap^TM^  2. Ethical approval was granted by a single university and reciprocal ethical approval was easily sought. Study was within the same country  3. Reducing study period due to resource constraints by analyzing previous studies in the field of interest | 1. Majority of participating teams have no research support in Sub-Saharan Africa |
| Mohan et al, 2021 (34) | Cross-sectional study | 2019 | [NIHR Global Health Research Group on Neurotrauma](https://pubmed.ncbi.nlm.nih.gov/?term=NIHR+Global+Health+Research+Group+on+Neurotrauma%5BCorporate+Author%5D),  National Institute for Health Research | Africa, Europe, South America, Asia | Nigeria, Ethiopia, Rwanda | 60 | 15 | Neurosurgery, Trauma Surgery | Traumatic brain injury | 1. Use of personal emails and social media platforms (Twitter, Facebook, and WhatsApp) | 1. Lack of internet access for those in rural and resource poor areas |
| Moysidis et al, 2021 (35) | Retrospective, Interventional, Case series | 2017-2019 | The Global Consortium | Africa, North America, Asia, Europe | South Africa, Egypt | 33 | 31 | Ophthalmology | Autologous Rental Transplantation | 1. Recruitment through multiple open invitations through  associations, in meetings, and via emails | 1. Scanty data for retrospective studies in LMCIs |
| Network for Peri-operative Critical care, 2021 (36) | Prospective, observational cohort | 2019-2020 | N4PCc,  Network for Peri-operative Critical care | Africa, Asia | Ethiopia | 4 | 28 | Perioperative care | Implementation of a perioperative registry | 1. Use of mobile applications with offline capability  2.Team training: online, quick reference guides,  3. Follow-up through instant messaging  4. Weekly meetings between collaborators via video conferencing  5. Information system with automated data analysis & live feedback  6. Single ethical review needed for the study: single country  7. Research mentors with experience in resource constraint setting  8. Funding secured from National Institute of Health Research and the UK Research Council | 1. Need for capacity building of the team  2. Lack of access to internet in some areas  3. Need for consistent follow-up |
| Oesophago-Gastric Anastomotic Audit Collaborative, 2021 (37) | Prospective, observational cohort | 2018 | OGAA,  [Oesophago-Gastric Anastomotic Audit (OGAA) Collaborative](https://pubmed.ncbi.nlm.nih.gov/?term=Oesophago-Gastric+Anastomotic+Audit+%28OGAA%29+Collaborative%3A+Writing+Committee%5BCorporate+Author%5D) | Africa, Europe, North America, South America, Asia, Oceania | Rwanda, Kenya, Nigeria | 141 | 613 | General surgery, Upper GI surgery | Mortality from esophagectomy in esophageal ca | 1. Data input via a secure information system i.e., REDCap^TM^ | 1. Each local site needed to seek its own ethical approval  2. Consultant or attending surgeon needed at each site to supervise data collection. These specialists are limited in LMCIs |
| PaedSurg Africa Research, 2021 (38) | Prospective, observational cohort | 2016-2017 | [PaedSurg Africa Research Collaboration](https://pubmed.ncbi.nlm.nih.gov/?term=PaedSurg+Africa+Research+Collaboration%5BCorporate+Author%5D) | Africa, Europe | Kenya, Nigeria, Ghana, Mauritania, DRC, South Africa, Burkina Faso, Côte d'Ivoire, Congo, Ethiopia, Malawi, Niger, Sudan, Tanzania, Zimbabwe, Zambia, Uganda, Cameroon, Ghana | 51 | 226 | Paediatric Surgery | Paediatric surgical conditions | 1. Recruitment through multiple channels: professional organizations, conferences, presentations, social media, and country leads  2. Data input via a secure information system i.e., REDCap^TM^  3. Study protocols & data collection in multiple languages  4. Initial pilot study conducted in a smaller number of centres to optimize study design  5. Local investigators individually followed up and contacted | 1. Limiting scope of study due to feasibility reasons  2. Participation by only 19 of 48 Sub-Saharan African (SSA) countries |
| Sanz Cortes et al, 2021 (39) | Prospective, observational cohort | 2013-2019 | International Fetoscopic Neural Tube Defect Repair Consortium | Africa, North America, South America, Europe, Asia, Middle East | South Africa | 14 | 20 | Paediatric Surgery | Fetoscopic surgery | 1. Data input via a secure information system i.e., REDCap^TM^ | Not described |
| Biccard et al, 2020 (40) | Delphi study | 2019 | APORG,  [African Peri-operative Research Group (APORG) working group](https://pubmed.ncbi.nlm.nih.gov/?term=African+Peri-operative+Research+Group+%28APORG%29+working+group%5BCorporate+Author%5D) | Africa, Europe, North America | South Africa, Uganda, Nigeria, Ghana, Botswana, Kenya, Mozambique, Tanzania, Ivory Coast, Ethiopia, Eswatini, Namibia, Sierra Leone, Botswana, Liberia, Egypt, Zambia, Senegal, Congo, Madagascar, Mali, Benin | 37 | 38 | Perioperative care | Perioperative research | 1. Use of multiple languages: English & French  2. Hybrid participation through physical and online meetings  3. Single ethical approval needed  4. Secured funding from Discipline of Anesthesiology and Critical Care, University of KwaZulu-Natal. | 1. Poor response rate of African clinicians |
| Brenner et al, 2020 (41) | Meta-analysis | N/A | [CRASH-3 trial collaborators](https://pubmed.ncbi.nlm.nih.gov/?term=CRASH-3+trial+collaborators%5BCorporate+Author%5D),  Corticosteroid randomisation after significant head injury | Africa, North America, South America, Europe, Asia | Cameroon, Nigeria, Kenya, Zambia, | 190 | 514 | Neurosurgery, Trauma Surgery | Improving outcome of traumatic brain injury | Not described | Not described |
| Chu et al, 2020 (42) | Expert opinion | 2020 | [AfroSurg Collaborative](https://pubmed.ncbi.nlm.nih.gov/?term=AfroSurg+Collaborative%5BCorporate+Author%5D) | Africa, North America | South Africa | 4 | 18 | Health system management | Access to safe surgery in Africa | Not described | 1. Funding disruptions during COVID-19  2. Disruptions in healthcare delivery due to COVID-19 |
| Delisle et al, 2020 (43) | Retrospective, observational | 2014-2016 | SOS & GlobalSurg,  [Surgical Outcomes Study Groups and GlobalSurg Collaborative](https://pubmed.ncbi.nlm.nih.gov/?term=Surgical+Outcomes+Study+Groups+and+GlobalSurg+Collaborative%5BCorporate+Author%5D) | Africa, Europe, North America | Algeria, Benin, Burundi, Cameroon, Congo, DRC, Egypt, Ethiopia, Gambia, Ghana, Kenya, Libya, Madagascar, Mali, Mauritius, Namibia, Niger, Nigeria, South Africa, Tanzania, Togo, Uganda, Zambia, Zimbabwe | 1464 | 3068 | General Surgery, Anaesthesia, Health system management | Access to safe surgery in Africa | Not described | 1. The underrepresentation of LMICs |
| Edlmann et al, 2020 (44) | Systematic review | 2019 | iCORIC,  [International Collaborative Research Initiative on Chronic Subdural Haematoma (iCORIC) study group](https://pubmed.ncbi.nlm.nih.gov/?term=International+Collaborative+Research+Initiative+on+Chronic+Subdural+Haematoma+%28iCORIC%29+study+group%5BCorporate+Author%5D) | Europe, Asia, South America, Africa | Ethiopia | N/A | 27 | Neurosurgery | Chronic subdural hematoma | Not described | Not described |
| EuroSurg et al, 2020 (45) | Prospective, observational cohort | 2020 | [EuroSurg Collaborative](https://pubmed.ncbi.nlm.nih.gov/?term=EuroSurg+Collaborative%5BCorporate+Author%5D) | Africa, Europe, Oceania | South Africa | 15 | 68 | General Surgery, Colorectal surgery | International surgical clinical audits | 1. Use of mini teams  2. Having a local senior consultant supervise junior investigators  3. Use of online information system REDCap^TM^  4. Management group with a support advisory group employed to coordinate the collaborative  5. All collaborators trained using an online e-learning package  6. Use of independent collaborators to provide data validation | 1. Ethical approval sought independently for each region |
| Ford et al, 2020 (46) | Retrospective, Cross-sectional observational study | 2016-2017 | OxPLORE Collaboration,  Oxford Pediatrics Linking Oncology Research with Electives | Africa, Europe, North America | Tanzania, Rwanda | 3 | 13 | Paediatric Surgery | Student led research collaboration; Wilms’ tumour | 1. Recruitment of investigators through advertising events  2. Student endorsed by a senior author to facilitate obtaining ethical approval  3. Use of electronic messaging and conferences to enable continued communication  4. Funding was sourced and made available  5. Use of medical students as collaborators overcomes the issue of limited research time | 1. Few qualified researchers in LMICs  2. Low availability of funding in LMICs  3. Lack of protected research time for LMICs collaborators  4. Power imbalance between HIC & LMICs in developing a collaborative research agenda  5. Limited infrastructure in LMICs  6. Missing data for retrospective review in LMICs |
| GlobalSurg et al, 2020 (47) | Prospective, observational cohort | 2016 | [GlobalSurg Collaborative](https://pubmed.ncbi.nlm.nih.gov/?term=GlobalSurg+Collaborative%5BCorporate+Author%5D) | Africa, Europe, Asia, North America, South America, Oceania | Egypt, Ethiopia, Ghana, Nigeria, Sierra Leone, South Africa, Zambia, Burundi, Botswana, Malawi, Morocco, Rwanda, Kenya | 181 | 1776 | Paediatric Surgery | Surgical Site Infections in children | 1. Structured into teams of local investigators coordinated by a national lead investigator  2. A central steering committee to analyse the data  3. Each team had at least 1 consultant or attending-level surgeon  3. Compulsory training of the investigators  4. Recruitment via the GlobalSurg network, social media, and personal contacts  5. Use of a well-established published protocol  6. Use of an information system to collect data i.e., REDCap^TM^ | Not described |
| Held et al, 2020 (48) | Delphi study | 2018-2019 | LION,  [Learning Innovation via Orthopaedic Networks (LION) Group](https://pubmed.ncbi.nlm.nih.gov/?term=Learning+Innovation+via+Orthopaedic+Networks+%28LION%29+Group%5BCorporate+Author%5D) | Africa, Europe, North America | South Africa, Ghana, Malawi, Tanzania, Kenya, Namibia | N/A | 43 | Orthopaedics | Orthopaedic training in Southern Africa | Not described | Not described |
| Lynch et al, 2020 (49) | Cross-section study, focus group, survey | 2019 | ARGO,  [African Research Group for Oncology Collaborative](https://pubmed.ncbi.nlm.nih.gov/?term=African+Research+Group+for+Oncology+Collaborative%5BCorporate+Author%5D) | Africa, North America | Nigeria | 22 | 18 | Oncology | Oncology research, Breast biopsy | Not described | 1. Choosing participant from a single symposium presents a risk of sampling bias |
| Muhly et al, 2020 (50) | Systematic review | 2018-2019 | PPOG,  [Pediatric Perioperative Outcomes Group](https://pubmed.ncbi.nlm.nih.gov/?term=Pediatric+Perioperative+Outcomes+Group%5BCorporate+Author%5D) | Africa, North America, Oceania, Europe, Asia, | South Africa | N/A | 21 | Perioperative care | Paediatric Perioperative outcomes | Not described | Not described |
| Pindyck et al, 2020 (51) | Prospective, observational cohort | 2012-2016 | [African Intussusception Surveillance Network](https://pubmed.ncbi.nlm.nih.gov/?term=African+Intussusception+Surveillance+Network%5BCorporate+Author%5D) | Africa, North America | Ethiopia, Ghana, Kenya, Malawi, Tanzania, Zambia, Zimbabwe | 29 | 24 | Paediatric Surgery | Intussusception | Not described | Not described |
| Protopapas et al, 2020 (52) | Cross-sectional survey | 2020 | [COVID-19 International Congenital Heart Surgery Taskforce](https://pubmed.ncbi.nlm.nih.gov/?term=COVID-19+International+Congenital+Heart+Surgery+Taskforce%5BCorporate+Author%5D) | Africa, North America, South America, Europe, Asia | Ghana, South Africa | 176 | 35 | Cardiac Surgery, Paediatric Surgery | Impact of COVID-19 on Paediatric Cardiac Surgery | 1. Online recruitment of participants and distribution of survey  2. Collaboration with associations & organizations with a network of expert members | Not described |
| Robertson et al, 2020 (53) | Cross-sectional survey | 2018 | [WFNS Young Neurosurgeons Committee](https://pubmed.ncbi.nlm.nih.gov/?term=WFNS+Young+Neurosurgeons+Committee%5BCorporate+Author%5D),  World Federation of Neurosurgical Societies Young Neurosurgeons Committee | Africa, North America, South America, Europe, Asia, Middle East | Rwanda, Morocco, Algeria, Ethiopia, Cameroon | N/A | 23 | Neurosurgery | Neurosurgery training | 1. Recruitment through electronic mailing lists of continental and various neurosurgical societies, e-mail to personal contacts, and social media platforms (Twitter, Facebook, and WhatsApp)  2. Partnership between HICs and LMICs  3. Research funding availability | 1. The underrepresentation of those without reliable Internet, electronic devices, and e-mail are less likely to be captured  2. Use of English language only  3. Low access to research resources e.g., journals in LMICs  4. Minimal research training of clinicians from LMICs |
| McMeekin et al, 2020 (54) | Randomized Control Trial (RCT) | 2010-2015 | [OVIVA collaborators](https://pubmed.ncbi.nlm.nih.gov/?term=OVIVA+collaborators%5BCorporate+Author%5D),  Oral versus Intravenous Antibiotics for Bone and Joint Infection | Africa, Europe | Kenya | N/A | 11 | Orthopaedics | Antibiotics for Orthopaedics infections | Not described | Not described |
| Robertson et al, 2020 (55) | Cross-sectional survey | 2019 | [Collaborative Working Group](https://pubmed.ncbi.nlm.nih.gov/?term=Collaborative+Working+Group%5BCorporate+Author%5D) | Africa, North America, South America, Europe, Asia, Middle East, Oceania | Cameroon, Malawi, South Africa, Rwanda | 47 | 111 | Neurosurgery | Task shifting and sharing in neurosurgery in LMIC | 1. Wide distribution of survey via electronic mailing lists of continental societies and various other neurosurgical groups, e-mail to personal contacts, QR codes, and social media platforms through | 1. Challenge reaching clinicians with limited access to internet |
| Papadopoulos et al, 2020 (56) | Case series | 2019 | CGRN,  [International Study of Childhood Glaucoma – Childhood Glaucoma Research Network Study Group](https://pubmed.ncbi.nlm.nih.gov/?term=International+Study+of+Childhood+Glaucoma+%E2%80%93+Childhood+Glaucoma+Research+Network+Study+Group%5BCorporate+Author%5D) | Africa, North America, Europe, Asia, Middle East | Ghana | 17 | 32 | Ophthalmology | Childhood glaucoma | 1. Use of an online data collection system  2. Recruitment of a large pool of international specialist using online methods | 1. Each centre was required to obtain Institutional Review Board (or equivalent) approval and parental/caregiver consent when required  2. Dominance of 2 centres in a particular region contributing 60% of patients enrolled  3. International variations in definitions and treatments making the study results difficult to standardize and interpret |
| Brenner et al, 2019 (57) | RCT | 2012-2018 | [HALT-IT Trial Collaborators](https://pubmed.ncbi.nlm.nih.gov/?term=HALT-IT+Trial+Collaborators%5BCorporate+Author%5D),  Haemorrhage Alleviation with Tranexamic acid – Intestinal system | Africa, North America, Europe, Asia | Nigeria, Egypt, Sudan, | 164 | 287 | General Surgery | RCT on GIT bleeding and tranexamic acid | Not described | 1. Ethical approval sought from national and local research ethics committees of participating countries |
| A. E.-A. H. A. R. C. S. M. group et al, 2019 (58) | Retrospective, observational | 2000-2016 | E-AHPBA,  [European-African HepatoPancreatoBiliary Association (E-AHPBA) Research Collaborative Study management group;](https://pubmed.ncbi.nlm.nih.gov/?term=A+European-African+HepatoPancreatoBiliary+Association+%28E-AHPBA%29+Research+Collaborative+Study+management+group%5BCorporate+Author%5D) | Europe, Africa | South Africa | 52 | 32 | General Surgery; Hepatobiliary surgery | Hepatobiliary disease | Not described | 1. Ethical approval sought from national and local research ethics committees of participating countries  2. Some centres contributed larger number of patients therefore risk of skewing data  3. Retrospective study design limitations i.e., selection bias, short follow-up |
| Dewan et al, 2019 (59) | Mixed-Method cross-sectional survey and expert opinion | N/A | PGSS,  Program in Global Surgery and Social Change collaborators | Africa, North America, Europe | South Africa, Uganda | 130 | 50 | Neurosurgery | Access to safe neurosurgery in LMCI | Not described | Not described |
| GlobalSurg, 2019 (60) | Meta-analysis | 2014-2016 | GlobalSurg Collaborative | Africa, North America, South America, Europe, Asia, Oceania, Middle East | Benin, Cameroon, Egypt, Ethiopia, Ghana, Libya, Malawi, Mozambique, Nigeria, Rwanda, South Africa, Tanzania, Zambia, Sudan | 242 | 3427 | General Surgery | Colostomy and colorectal resection | 1. Well defined structure for the collaborative | 1. Low representation of LMICs. Approximately 25% of the data |
| GlobalSurg, 2019 (61) | Meta-analysis | 2014-2016 | [GlobalSurg Collaborative](https://pubmed.ncbi.nlm.nih.gov/?term=GlobalSurg+Collaborative%5BCorporate+Author%5D) | Africa, North America, South America, Europe, Asia, Oceania, Middle East | Benin, Botswana, Burundi, Cameroon, Egypt, Ethiopia, Ghana, Libya, Kenya, Madagascar, Morocco, Malawi, Mozambique, Nigeria, Rwanda, South Africa, Sudan, Tanzania, Zambia | 356 | 3445 | General Surgery | WHO surgical safety checklist use audit | 1. Well defined structure for the collaborative | 1. Better resourced institutions have a higher number of collaborators  2. Data validation by independent teams is difficult in resource limited institutions |
| Iverson et al, 2019 (62) | Mixed-Method descriptive qualitative and quantitative study | 2016 | [Safe Surgery 2020 Collaborators](https://pubmed.ncbi.nlm.nih.gov/?term=Safe+Surgery+2020+Collaborators%5BCorporate+Author%5D) | Africa, America | Ethiopia | 15 | 13 | Health system management | Access surgical capacity | 1. Translation of research tools to local language | 1. Low number of surgical specialists in Ethiopia |
| Li et al, 2019 (63) | RCT | 2010-2015 | [OVIVA Trial Collaborators](https://pubmed.ncbi.nlm.nih.gov/?term=OVIVA+Trial+Collaborators%5BCorporate+Author%5D),  Oral versus Intravenous Antibiotics for Bone and Joint Infection | Africa, Europe | Kenya | 26 | 53 | Orthopaedics | Antibiotics for Orthopaedic infections | Not described | Not described |
| Robertson et al, 2019 (64) | Cross-sectional survey | 2018-2019 | [Global Neurosurgery Survey Collaborators](https://pubmed.ncbi.nlm.nih.gov/?term=Global+Neurosurgery+Survey+Collaborators%5BCorporate+Author%5D) | Africa, North America, South America, Europe, Asia, Middle East, Oceania | Cameroon, Malawi, South Africa, Rwanda | 105 | 245 | Neurosurgery | Task shifting and sharing in neurosurgery | 1. Research tools translated into 3 languages: English, French, & Spanish  2. Wide distribution of survey via electronic mailing lists of continental societies and various other neurosurgical groups, e-mail to personal contacts, QR codes, and social media platforms | 1. Difficulty achieving ideal distribution ratios between HICs & LMCIs, as well as between rural & urban regions |
| Fink et al, 2018 (65) | Prospective, observational cohort | N/A | [Pediatric Acute Lung Injury and Sepsis Investigators (PALISI) Network, PALISI Global Health Subgroup, and Prevalence of Acute Critical Neurological Disease in Children: A Global Epidemiological Assessment (PANGEA) Investigators](https://pubmed.ncbi.nlm.nih.gov/?term=Pediatric+Acute+Lung+Injury+and+Sepsis+Investigators+%28PALISI%29+Network%2C+PALISI+Global+Health+Subgroup%2C+and+Prevalence+of+Acute+Critical+Neurological+Disease+in+Children%3A+A+Global+Epidemiological+Assessment+%28PANGEA%29+Investigators%5BCorporate+Author%5D),  Pediatric Acute Lung Injury and Sepsis Investigators, Prevalence of Acute Critical Neurological Disease in Children: A Global Epidemiological Assessment Investigators | Africa, North America | Kenya, Ethiopia, Rwanda, Ghana | 4 | 23 | Paediatric Surgery, Neurosurgery | Epidemiology and outcome of TBI & infectious encephalopathy | Not described | 1. Ethical approval from each enrolled centre |
| GlobalSurg, 2018 (66) | Prospective, observational cohort | 2016 | [GlobalSurg Collaborative](https://pubmed.ncbi.nlm.nih.gov/?term=GlobalSurg+Collaborative%5BCorporate+Author%5D) | Africa, North America, South America, Europe, Asia, Oceania, Middle East | Benin, Botswana, Burundi, Egypt, Ethiopia, Ghana, Madagascar, Malawi, Nigeria, South Africa, Sudan, Zambia | 343 | 1816 | General Surgery | Surgical site infection after GI surgery | 1. Structured into teams of local investigators coordinated by a national lead investigator  2. Recruitment via the GlobalSurg network, social media, and personal contacts  3. Use of a well-established published protocol  4. Use of an information system to collect data i.e., REDCap^TM^  5. The study received funding | 1. Individual ethical approval needed for multiple sites |
| Goodman et al, 2018 (67) | Expert working group | 2016 | [GICS Collaborators](https://pubmed.ncbi.nlm.nih.gov/?term=GICS+Collaborators%5BCorporate+Author%5D),  Global Initiative for Children's Surgery | Africa, North America, Europe | Nigeria | 76 | 145 | Paediatric Surgery | Optimizing children's surgical care in LMIC | Not described | 1. Limited number and diversity of participants |
| Israels, et al, 2018 (68) | Prospective, observational cohort | 2013 | the Collaborative Wilms Tumour Africa team | Africa, Europe | Malawi, Cameroon, Ghana, Zimbabwe, Uganda, Ethiopia | 8 | 14 | Paediatric Surgery | Wilms’ Tumour | 1. Having multidisciplinary stakeholders has strengthen collaboration  2. Having dedicated full time data managers | 1. Investigators have other duties & lack dedicated research time to input, clean, or analyse data |
| Tate et al, 2018 (69) | Case series | 2012-2016 | [African Intussusception Surveillance Network](https://pubmed.ncbi.nlm.nih.gov/?term=African+Intussusception+Surveillance+Network%5BCorporate+Author%5D) | Africa, North America, Europe | Conga, Ethiopia, Ghana, Kenya, Malawi, Tanzania, Zambia, Zimbabwe | 29 | 26 | Paediatric Surgery | Intussusception | Not described | Not described |
| Reilingh et al, 2018 (70) | Delphi study | 2017 | [International Consensus Group on Cartilage Repair of the Ankle](https://pubmed.ncbi.nlm.nih.gov/?term=International+Consensus+Group+on+Cartilage+Repair+of+the+Ankle%5BCorporate+Author%5D) | Africa, Europe, North America, Asia, Oceania | South Africa | 25 | 85 | Orthopaedics | Ankle repair surgery | Not described | Not described |
| Van Dijk et al, 2018 (71) | Delphi study | 2017 | [International Consensus Group on Cartilage Repair of the Ankle](https://pubmed.ncbi.nlm.nih.gov/?term=International+Consensus+Group+on+Cartilage+Repair+of+the+Ankle%5BCorporate+Author%5D) | Africa, Europe, North America, Asia, Oceania | South Africa | 25 | 84 | Orthopaedics | Ankle repair surgery | Not described | Not described |
| Sprague et al, 2018 (72) | Prospective, observational cohort | 2011-2018 | [INORMUS Investigators](https://pubmed.ncbi.nlm.nih.gov/?term=INORMUS+Investigators%5BCorporate+Author%5D),   International Orthopaedic Multicentre Study | Africa, South America, North America, Asia, Oceania | Uganda, Kenya, Tanzania, South Africa, Nigeria, Botswana, Ghana | 50 | 146 | Orthopaedics | Musculoskeletal trauma in LMCIs | 1. Conducting a pilot study to improve study design and feasibility  2. Secure funding for the study  3. Use of existing relationships & networks  4. Establishing regional leads/ champions  5. Establishing a central investigative team with established researchers to facilitate  6. Research staff fluent in local languages  7. Having a clearly defined structure | 1. Difficulty recruiting investigators in regions where relationships and networks of the collaborators were poor  2. Understanding local policy, culture, and other context of different regions |
| Dresser et al, 2017, (73) | Retrospective, observational | 2011-2014 | [Global Emergency Care Collaborative Investigators](https://pubmed.ncbi.nlm.nih.gov/?term=Global+Emergency+Care+Collaborative+Investigators%5BCorporate+Author%5D) | Africa, North America | Uganda | 1 | 6 | General Surgery | Emergency surgical care | Not described | Not described |
| Brink et al, 2017 (74) | Prospective, observational cohort | 2013-2015 | NASSA,  [Netcare Antimicrobial Stewardship Study Alliance](https://pubmed.ncbi.nlm.nih.gov/?term=Netcare+Antimicrobial+Stewardship+Study+Alliance%5BCorporate+Author%5D) | Africa | South Africa | 34 | 346 | Perioperative care | Peri-operative antibiotic use | 1. Single ethical approval  2. Training sessions for all participants  3. Multidisciplinary collaborative team | 1. Variation of processes & implementation in-between centres. This additionally, made centre to centre comparisons difficult. |
| Ekure et al, 2017 (75) | Cross-sectional survey | 2014 | [Nigerian Pediatric Cardiology Study Group](https://pubmed.ncbi.nlm.nih.gov/?term=Nigerian+Pediatric+Cardiology+Study+Group%5BCorporate+Author%5D) | Africa | Nigeria | 17 | 31 | Paediatric Surgery; Cardiothoracic surgery | Paediatric Heart Disease/ Congenital Heart Defects | 1. Single ethical approval required as all centres were in the same country | 1. No financial support to the authors |
| Czauderna et al, 2016 (76) | Retrospective, observational | 2011 | CHIC,  The Children's Hepatic Tumors International Collaboration | Africa, North America, Europe, Asia | Malawi | 9 | 20 | Paediatric Surgery | Children hepatic tumours | 1. Creation of a web portal to facilitate communication, information sharing, and working online  2. Establishing a steering committee to govern the collaboration  3. Frequent formal and informal meetings  4. Open sharing of information and ideas which helped to build trust  5. Developing a clear framework for the collaboration  6. Augmenting modern communication tools with traditional face to face meetings | 1. Variability of retrospective data |
| Yang et al, 2016 (77) | Retrospective, observational | 2006-2016 | [Africa Network for Gastrointestinal and Liver Diseases](https://pubmed.ncbi.nlm.nih.gov/?term=Africa+Network+for+Gastrointestinal+and+Liver+Diseases%5BCorporate+Author%5D) | Africa, North America, | Cameroon, Egypt, Ethiopia, Ghana, Ivory Coast, Nigeria, Sudan, Tanzania, Uganda | 21 | 177 | General Surgery | Hepatocellular carcinoma in Africa | Not described | 1. Shortage of medical resources  2. Scanty data for retrospective studies in LMCIs  3. Difficulty getting participants from all the regions in Africa i.e., Southern African not represented |
| Mwinga, et al, 2015 (78) | Cross-sectional survey | 2012 | [SIRCLE Collaboration](https://pubmed.ncbi.nlm.nih.gov/?term=SIRCLE+Collaboration%5BCorporate+Author%5D) | Africa, North America, Europe | Kenya | 22 | 16 | Health system management | Quality of surgical care | 1. 1 week training and a pilot survey prior to the start of the study  2. Division of the investigators into small teams of 4-5 members lead by an experienced researcher  3. Use of an information system to collect data i.e., REDCap^TM^  4. Funding from Kenya Medical Research Institute (KEMRI)-Welcome Trust | Not described |

**Highlighted link to the original publication*

**Supplementary Table 4** Summary of study designs

| **Study design** | **Number of studies** | **Percentage** |
| --- | --- | --- |
| Prospective, observational cohort | 14 | 25% |
| Delphi & Expert opinion | 10 | 18% |
| Cross-sectional survey | 8 | 15% |
| Retrospective, observational | 8 | 15% |
| Mixed method | 5 | 9% |
| Meta-analysis | 3 | 5% |
| RCT | 3 | 5% |
| Case series | 2 | 4% |
| Systematic review | 2 | 4% |
| **TOTAL** | **55** | **100%** |

**Supplementary Table 5:** Summary of participation in collaborative surgical publications in Africa (by region)

|  | **Country** | **Studies** |
| --- | --- | --- |
| **Northern Africa** |  | **30** |
|  | Egypt | 12 |
|  | Sudan | 6 |
|  | Algeria | 4 |
|  | Libya | 4 |
|  | Morocco | 4 |
|  | Tunisia | 0 |
|  |  |  |
| **Eastern Africa** |  | **114** |
|  | Ethiopia | 19 |
|  | Kenya | 18 |
|  | Malawi | 12 |
|  | Tanzania | 12 |
|  | Rwanda | 11 |
|  | Uganda | 11 |
|  | Zambia | 11 |
|  | Zimbabwe | 7 |
|  | Burundi | 5 |
|  | Madagascar | 5 |
|  | Mozambique | 3 |
|  | Comoros | 0 |
|  | Djibouti | 0 |
|  | Eritrea | 0 |
|  | Mauritius | 0 |
|  | Seychelles | 0 |
|  | Somalia | 0 |
|  | South Sudan | 0 |
|  |  |  |
| **Central Africa** |  | **17** |
|  | Cameroon | 12 |
|  | Congo, DRC | 5 |
|  | Angola | 0 |
|  | Central African Republic | 0 |
|  | Chad | 0 |
|  | Equatorial Guinea | 0 |
|  | Gabon | 0 |
|  | São Tomé and Príncipe | 0 |
|  |  |  |
| **Southern Africa** |  | **40** |
|  | South Africa | 29 |
|  | Botswana | 5 |
|  | Namibia | 5 |
|  | Eswatini (Swaziland) | 1 |
|  | Lesotho | 0 |
|  |  |  |
| **Western Africa** |  | **65** |
|  | Nigeria | 21 |
|  | Ghana | 17 |
|  | Benin | 6 |
|  | Niger | 4 |
|  | Mali | 3 |
|  | Senegal | 3 |
|  | Côte d'Ivoire (Ivory Coast) | 2 |
|  | Gambia | 2 |
|  | Liberia | 2 |
|  | Sierra Leone | 2 |
|  | Togo | 2 |
|  | Burkina Faso | 1 |
|  | Cape Verde | 0 |
|  | Guinea | 0 |
|  | Guinea-Bissau | 0 |
|  | Mauritania | 0 |
|  | Saint Helena, Ascension, and Tristan da Cunha | 0 |

**Supplementary Table 6** Statistics of the surgical specialties from the studies reviewed

| **Surgical specialty** | **Studies** | **Percentage** |
| --- | --- | --- |
| Neurosurgery | 14 | 17% |
| Paediatric | 14 | 17% |
| General surgery | 12 | 14% |
| Orthopaedics | 10 | 12% |
| Trauma | 8 | 10% |
| Health systems management | 6 | 7% |
| Perioperative care | 6 | 7% |
| Cardiac | 3 | 4% |
| Anaesthesia | 2 | 2% |
| Colorectal | 2 | 2% |
| Hepatobiliary | 2 | 2% |
| Oncology | 2 | 2% |
| Ophthalmology | 2 | 2% |
| Upper GI | 1 | 1% |
| **Total no. of specialties** | **14** |  |
| **Maximum per collaborative** | **14** |  |
| **Minimum per collaborative** | **1** |  |
| **Average per collaborative** | **6** |  |

**Supplementary Table 7:** List of Facilitating factors & challenges affecting collaborative research studies

| **Supplementary Table 7a:** List of Facilitating Factors affecting surgical collaboratives | | | |
| --- | --- | --- | --- |
|  | | | |
| **Category** | | **Facilitating factors** | **No. of studies** |
| **Information & communication** | | | **43** |
|  | | Use of online tools, data capture, information systems, & resources to share & store information | 16 |
|  | | Maximizing recruitment through multiple channels: social media, multimedia (videos), personal emails, associations, networks & promotion/ advertising the studies and aims | 10 |
|  | | Use of local language/ multiple languages | 6 |
|  | | Maintaining regular communication & reminders with collaborators- emails, texts, meetings | 5 |
|  | | Using hybrid communication & data collection systems that combine both paper, physical, off-line capable devices, with online tools | 4 |
|  | | Open sharing of information to build trust | 1 |
|  | | Scheduling international online meetings/ forums at convenient time for all | 1 |
| **Structure & Design** | | | **37** |
|  | | Structured mini teams with local leadership/ champions for easy follow-up & communication to teams | 6 |
|  | | Training and orientation of investigators | 6 |
|  | | Clear structure & terms of reference for the collaborators | 5 |
|  | | Having a central management team to govern, steer, and coordinate the collaborative | 5 |
|  | | Having experienced research mentors, or consultants for juniors | 4 |
|  | | Having a diverse multidisciplinary team of collaborators | 3 |
|  | | Using an easily replicable, & established study method or protocol | 3 |
|  | | Local representation, such as having a majority of LMICs collaborators improves participation of individuals from these regions | 2 |
|  | | Setting clear goals & targets e.g. No of surveys per day | 1 |
|  | | Use of independent collaborators for data verification | 1 |
|  | | Use of medical students as investigators especially where collaborators have limited time | 1 |
| **Resources** | | | **11** |
|  | | Secure & adequate financial resource planning | 8 |
|  | | Running smaller pilot studies to optimize study design, understand resource requirements, & feasibility | 2 |
|  | | Partnership between HIC, & LMICs to leverage synergies | 1 |
| **Ethics** | | | **6** |
|  | | Single country studies that require only 1 ethical approval for a multicentre study | 5 |
|  | | Identifying required ethical approvals for different regions early | 1 |
| **Network** | | | **4** |
|  | | Support from associations, groups, and networks | 2 |
|  | | Use of existing networks and relationships | 2 |
| **Supplementary Table 7b:** List of challenges to surgical collaboratives | | | |
| **Category** | **Challenges** | | **No. of studies** |
| **Structure & Design** | | | **32** |
|  | Difficulty in achieving desired distribution & diversity of collaborators. Underrepresentation/ small number of collaborators especially from LMICs, poor response rate of African collaborators, may lead to skewed distribution can lead to bias e.g., if HICs are over represented | | 8 |
|  | High variability of retrospective data, scanty & missing data in these studies especially in LMICs | | 4 |
|  | Lack of research training & experience of participants, especially from LMICs/ Africa | | 4 |
|  | Difficulty interpreting data from different regions e.g., variation in costs, context, processes | | 3 |
|  | Need for multi-stage/ round studies. This is further constrained by time, and drop out in participation as rounds continue | | 3 |
|  | Difficulty in achieving consensus between members | | 2 |
|  | Poor recruitment strategy can lead to bias or lack of sufficient collaborators | | 2 |
|  | Some proposals were beyond scope of the ongoing studies or collaborations | | 2 |
|  | No standardized approach to collaborative studies | | 1 |
|  | Power imbalance between LMICs and HICs collaborators in developing research agenda | | 1 |
|  | Some centres have larger patient numbers and can lead to skewed data | | 1 |
|  | Need for team capacity building/ training | | 1 |
| **Resources** | | | **19** |
|  | Need to have financial resources available e.g., LMICs have limited access to resources | | 7 |
|  | Poor research support for investigators in Africa e.g., Lack of protected research time, lack of access to journals | | 4 |
|  | Difficulty finding specialist in LMICs | | 3 |
|  | Low number of participating African countries | | 2 |
|  | Lack of first authors/ lead authors from LMICs | | 1 |
|  | Limited infrastructure in LMICs | | 1 |
|  | Limiting/ reducing scope of study due to lack of adequate financing | | 1 |
| **Information & communication** | | | **14** |
|  | Lack of internet access in some regions. Collaborators with poor access to internet and electronic devices may miss out | | 5 |
|  | Language challenges such as: (i) difficulty in translation, (ii) use of multiple languages | | 3 |
|  | Poor information systems, lack of access to online platforms, and cost of IT/ online systems | | 2 |
|  | Availability of online forums later as recorded videos, reduced active live engagement and collaboration in online forums | | 1 |
|  | Online interaction & webinar fatigue | | 1 |
|  | Need for consistent follow-up | | 1 |
|  | Need for understanding of multiple different local contexts, culture, policy etc. | | 1 |
| **Ethics** | | | **7** |
|  | Multiple ethical approvals needed for different regions resulting in additional costs, and delays | | 7 |
| **Network** | | | **1** |
|  | Need for personal networks and relationships to improve recruitment | | 1 |
| **Other** | | | **2** |
|  | COVID-19 disruption to funding and healthcare delivery | | 2 |
